# Optimizing quantification of MK6240 tau PET in unimpaired older adults

**DOI:** 10.1016/j.neuroimage.2022.119761

**Published:** 2022-11-28

**Authors:** Theresa M. Harrison, Tyler J. Ward, Alice Murphy, Suzanne L. Baker, Pablo A. Dominguez, Robert Koeppe, Prashanthi Vemuri, Samuel N. Lockhart, Youngkyoo Jung, Danielle J. Harvey, Laura Lovato, Arthur W. Toga, Joseph Masdeu, Hwamee Oh, Darren R. Gitelman, Neelum Aggarwal, Heather M. Snyder, Laura D. Baker, Charles DeCarli, William J. Jagust, Susan M. Landau

**Affiliations:** a University of California Berkeley, USA; b Lawrence Berkeley National Laboratory, USA; c University of Michigan, USA; d Mayo Clinic, USA; e Wake Forest University School of Medicine, USA; f University of California Davis, USA; g University of Southern California, USA; h Houston Methodist, USA; i Brown University, USA; j Advocate Aurora Health, USA; k Rush University Medical Center, USA; l Alzheimer’s Association, USA

**Keywords:** PET, Tau, Amyloid, Aging, Alzheimer’s disease, off-target signal, meninges

## Abstract

Accurate measurement of Alzheimer’s disease (AD) pathology in older adults without significant clinical impairment is critical to assessing intervention strategies aimed at slowing AD-related cognitive decline. The U.S. Study to Protect Brain Health Through Lifestyle Intervention to Reduce Risk (POINTER) is a 2-year randomized controlled trial to evaluate the effect of multicomponent risk reduction strategies in older adults (60-79 years) who are cognitively unimpaired but at increased risk for cognitive decline/dementia due to factors such as cardiovascular disease and family history. The POINTER Imaging ancillary study is collecting tau-PET ([^18^F]MK6240), beta-amyloid (A*β*)-PET ([^18^F]florbetaben [FBB]) and MRI data to evaluate neuroimaging biomarkers of AD and cerebrovascular pathophysiology in this at-risk sample. Here 481 participants (70.0±5.0; 66% F) with baseline MK6240, FBB and structural MRI scans were included. PET scans were coregistered to the structural MRI which was used to create FreeSurfer-defined reference regions and target regions of interest (ROIs). We also created off-target signal (OTS) ROIs to examine the magnitude and distribution of MK6240 OTS across the brain as well as relationships between OTS and age, sex, and race. OTS was unimodally distributed, highly correlated across OTS ROIs and related to younger age and sex but not race. Aiming to identify an optimal processing approach for MK6240 that would reduce the influence of OTS, we compared our previously validated MRI-guided standard PET processing and 6 alternative approaches. The alternate approaches included combinations of reference region erosion and meningeal OTS masking before spatial smoothing as well as partial volume correction. To compare processing approaches we examined relationships between target ROIs (entorhinal cortex (ERC), hippocampus or a temporal meta-ROI (MetaROI)) SUVR and age, sex, race, A*β* and a general cognitive status measure, the Modified Telephone Interview for Cognitive Status (TICSm). Overall, the processing approaches performed similarly, and none showed a meaningful improvement over standard processing. Across processing approaches we observed previously reported relationships with MK6240 target ROIs including positive associations with age, an A*β*+> A*β*- effect and negative associations with cognition. In sum, we demonstrated that different methods for minimizing effects of OTS, which is highly correlated across the brain within subject, produced no substantive change in our performance metrics. This is likely because OTS contaminates both reference and target regions and this contamination largely cancels out in SUVR data. Caution should be used when efforts to reduce OTS focus on target or reference regions in isolation as this may exacerbate OTS contamination in SUVR data.

## 1. Introduction

Quantification of early pathological tau in individuals without significant cognitive impairment is essential to uncovering the mechanisms by which Alzheimer’s disease (AD) pathology leads to cognitive decline (Jagust, 2018). Since 2014, several tau-PET tracers have been developed and used to label tau, allowing regional *in vivo* measurement of tau pathology burden (Groot et al., 2022; Schöll et al., 2019). This spatial information is critical as it has been shown that not only overall burden but the distribution of tau predicts specific cognitive and clinical outcomes (Bejanin et al., 2017; La Joie et al., 2020; Ossenkoppele et al., 2016; Pereira et al., 2020; Phillips et al., 2018; Xia et al., 2017). To date, tau-PET tracers have been used to localize and quantify tau in cognitively normal older controls, individuals with mild cognitive impairment (MCI) and individuals with AD or other neurodegenerative diseases where abnormal tau accumulation is a feature of the pathophysiology (Groot et al., 2022). The use of these tracers across the clinical spectrum is critical to understanding how AD pathology emerges and spreads, but each tau-PET tracer has known off-target binding leading to patterns of off-target signal (OTS) which makes quantification in some regions more challenging (Baker et al., 2019; Betthauser et al., 2019; Choi et al., 2018; Marquié et al., 2015; Smith et al., 2021).

Across several tracers, tau-PET OTS has been shown to be related to factors associated with AD incidence and progression including age, sex and race (Baker et al., 2019; Lee et al., 2018; Smith et al., 2021). This can complicate interpretation of tau-PET signal especially in regions adjacent to known OTS such as the hippocampus which lies just ventral to choroid plexus, a site of OTS with flortaucipir (Baker et al., 2017; Pawlik et al., 2020). In addition, most studies using tau-PET tracers, regardless of clinical status, have been completed in overwhelmingly white cohorts. Here, we have the unique opportunity to assess the relationship between OTS and key biological variables in a more diverse sample of participants that more closely resembles the population diversity of the United States. Our cohort are all participants from the U.S. Protect Brain Health Through Lifestyle Intervention to Reduce Risk (POINTER) study (uspointer.net), a lifestyle intervention clinical trial enrolling older adults without cognitive impairment who are at elevated risk for future decline.

MK6240 is a second-generation tau-PET tracer with several advantages over first-generation tracers, including minimal OTS in the basal ganglia and choroid plexus and a higher dynamic range, especially in individuals with AD with more advanced tau pathology (Betthauser et al., 2019; Pascoal et al., 2018; Walji et al., 2016). Like other tau-PET tracers, however, MK6240 shows OTS, particularly in the meninges, that may interfere with accurate quantification of early tau deposition (Betthauser et al., 2019). Early tau accumulation is critical to measure in individuals enrolled in the POINTER study, who are cognitively unimpaired, at risk for decline and in whom the *overall* burden of tau is expected to be low compared to individuals with AD. As MK6240 is a relatively new tracer, methods for processing these images and correcting for the influence of OTS are not established.

In the present study, we had two main aims. The first aim was to characterize MK6240 meningeal OTS in a large cross-sectional cohort of older adults from the POINTER Imaging ancillary study. This included describing key problems arising from meningeal signal and exploring the biological drivers of meningeal binding. Specifically, we test whether age, sex or race is related to the magnitude of OTS in the meninges. Second, we explored various processing approaches, including partial volume correction (PVC) (Baker et al., 2017; Rousset et al., 1998), to mitigate the effect of meningeal OTS on target cortical regions with the ultimate goal of identifying the best approach for analysis of MK6240 data in cognitively unimpaired older adults. We evaluated the effects of MK6240 processing methods on previously-reported relationships between cortical tau and increased beta-amyloid (A*β*), older age and female sex, with the assumption that the optimal method should maximize the ability to detect these associations.

## 2. Methods

### 2.1. Participants

Participants were enrolled in the POINTER Imaging study, an ancillary study of the POINTER clinical trial that is testing whether random assignment to either of two multidomain lifestyle interventions (focusing on nutrition, physical exercise, cognitive/social stimulation, health monitoring) that differ in format, intensity and accountability affects 2-year cognitive trajectory. POINTER trial participants are aged 60-79 years, lack significant memory impairment, are sedentary, report a sub-optimal diet, and are at risk for future cognitive decline based on family history of memory impairment, race or ethnicity, and/or other risk factors (U.S. POINTER Clinical Trial, 2019). Enrollment into the POINTER trial and the POINTER Imaging ancillary study is ongoing. For the current study, participants were additionally required to have completed baseline tau-PET, A *β*-PET and structural MRI scans. Currently, limited demographic (age, sex and race) and cognitive (Modified Telephone Interview for Cognitive Status [TICSm]) data from the main trial are available for analysis. The TICSm is a well-established test of general cognitive function designed to be administered over the phone and that is used in screening to assess eligibility for the POINTER trial (Welsh et al., 1993).

POINTER Imaging participants were recruited from the communities surrounding five sites: UC Davis, Wake Forest University School of Medicine, Rush University and Advocate Aurora Health (combined Chicago site), Baylor College of Medicine, Kelsey Research Foundation and Houston Methodist (combined Houston site) and Brown University / Merriam Hospital and Butler Hospital (combined New England/Rhode Island site). The complete baseline dataset from the POINTER parent trial, including neuroimaging data, will be made available when enrollment and dataset curation has concluded. Publicly available neuroimaging data will be accessible via LONI by any qualified investigator that has formally agreed to the POINTER Imaging data use agreement. The central Institutional Review Board (IRB) at Wake Forest University School of Medicine approved this study, with concurrence by local site IRBs. All participants provided written informed consent.

### 2.2. Image acquisition & preprocessing

Participants underwent baseline PET scanning sessions for tau ([ ^18^ F]MK6240) and A *β* ([^18^ F]florbetaben [FBB]) pathology and an MRI session that included a structural scan to assess brain morphology.

Structural MRI (MPRAGE) scans were acquired for each POINTER participant using identical parameters to the ADNI3 protocol: TR=2300ms, TE=min full echo, voxel size=1mm isotropic, FOV=256mm. For all participants, sMRI data were segmented into cortical and subcortical regions based on anatomical landmarks using FreeSurfer v7.1 (Desikan et al., 2006; Fischl and Dale, 2000).

To acquire tau-PET images, participants were injected with 5 mCi of the MK6240 tracer and scanned from 90-110 minutes post-injection (4 × 5 minute frames). To acquire A *β*-PET images, approximately 8 mCi of FBB was injected and 20-min, post-injection acquisition with 4 × 5 minute frames from 90-110 minutes was completed. FBB images were pre-processed according to a protocol based on ADNI to account for scanner differences, including scanner-specific reconstruction parameters, coregistering and averaging individual frames, reorienting static images to a standard image grid and smoothing to common 6mm^3^ resolution (ADNI, 2022; Joshi et al., 2009). Fully pre-processed FBB SUVR images were downloaded from LONI and intensity normalized using the whole cerebellum. MK6240 images were downloaded from LONI after individual frames were averaged and reorientation complete, but before spatial smoothing. Intensity normalization was done using inferior cerebellar gray matter as the reference region. Differential smoothing of the MK6240 data to uniform 6mm^3^ resolution was part of each of the candidate pipelines (see details below).

Tracer-specific PET ROIs were sampled using MRI-based native space FreeSurfer parcellations (Desikan et al., 2006). FBB global SUVRs were calculated as previously described and POINTER participants were classified as A *β*+ with a global (FreeSurfer-derived frontal, temporal, parietal and posterior cingulate ROIs; Mormino et al., 2009) SUVR >1.08 (18 CL), a threshold developed and validated in independent datasets using identical acquisition and analysis parameters (Royse et al., 2021).

### 2.3. Image processing: OTS ROI creation

The overarching goals of this project were to 1) characterize MK6240 OTS and 2) optimize MK6240 quantification in POINTER Imaging participants.

**Fig. 1.**
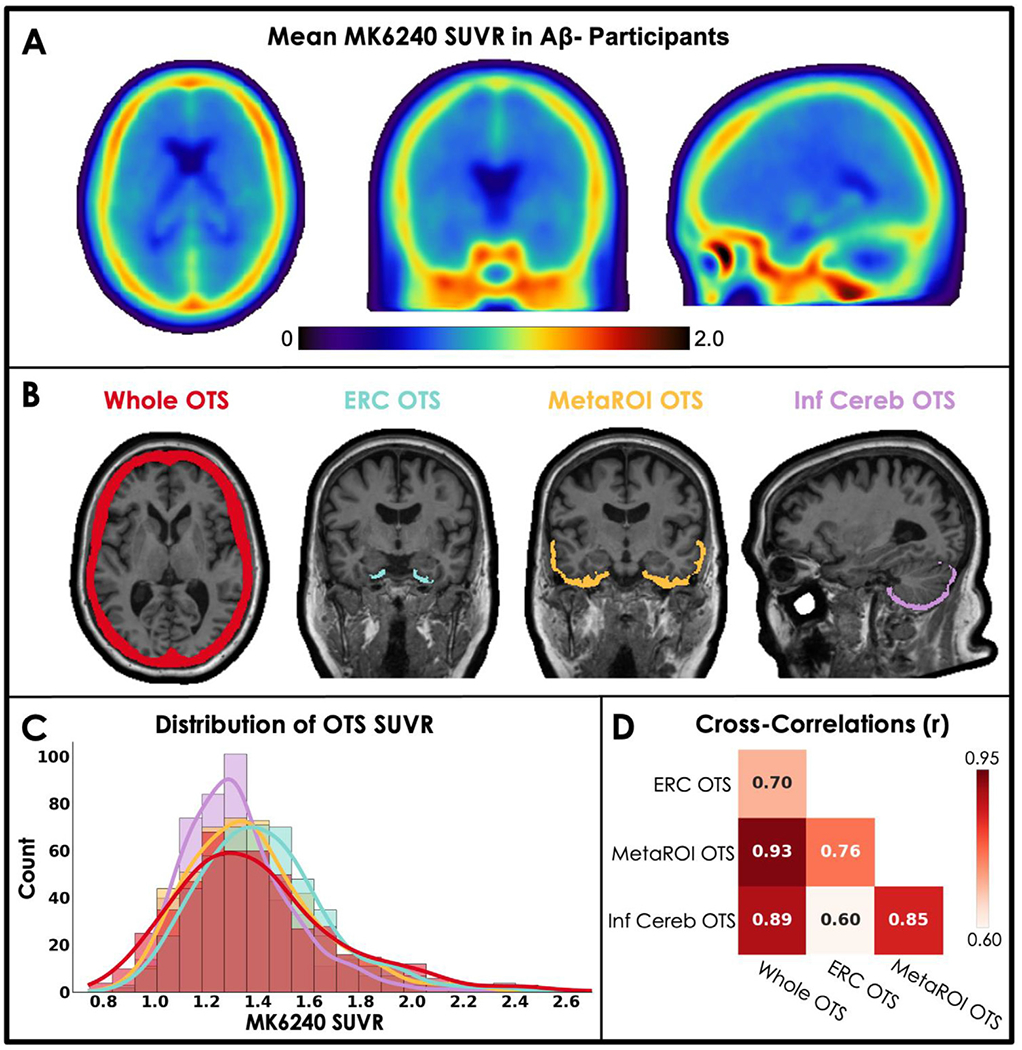
Quantifying off-target signal (OTS) near regions of interest (ROIs). (A) Mean voxel-wise average of A *β* negative standard uptake value ratio (SUVR) [18F]MK6240 binding pattern in MNI152 space (n=80). (B) OTS ROIs are shown overlaid on a participant MRI. The reverse-normalized whole OTS ROI is shown in red. Subject-specific OTS ROIs displayed are a subset of the voxels inside the whole OTS mask for regions bordering the entorhinal cortex (turquoise), temporal MetaROI (yellow), and inferior-cerebellar GM (purple). (C) The distribution of mean regional SUVRs for each OTS ROI (n=481). (D) Results from cross-Pearson-correlations within-subject OTS ROI SUVRs (all p < 0.001).

To accomplish the first goal, we created novel OTS ROIs to measure meningeal signal across the whole scan (‘whole OTS’) and adjacent to key regions including the entorhinal cortex (ERC), a meta temporal region often used to stage tau pathology (MetaROI; Jack et al., 2017) and the inferior cerebellar gray matter. The whole OTS mask was created by warping (‘Normalize’ in SPM12) A *β*-participants’ (n=80) MRI-coregistered MK6240 SUVR images into template space (T1w MNI-152 template provided by SPM12) and averaging the resulting template space MK6240 SUVR images (Fig. 1A). We then thresholded the average SUVR image at SUVR 1.16, which visually captured meningeal binding while excluding cortex, and created a binary mask. This thresholded mask in template space was then reverse-normalized into native space for each participant (n=481).

We used the whole OTS mask as an exclusive mask for MK6240 scans to exclude OTS signal in some of our candidate pipelines (described below) and also for quantifying OTS itself. In addition to measuring OTS in native space using the whole OTS mask created in template space, we created novel OTS ROIs defined in native space that were adjacent to the ERC, MetaROI and inferior cerebellar gray matter by first smoothing each subject-specific, FreeSurfer-defined region with a 6mm FWHM Gaussian kernel, thresholding at 0.05, and then capturing the overlap between the smoothed ROI and the reverse-normalized whole OTS mask. Importantly, when quantifying OTS, we excluded cortical voxels since we were only interested in signal outside the brain. All OTS ROI SUVRs were created by normalizing by inferior cerebellar gray.

### 2.4. Imaging processing: Target ROI selection

Another component of characterizing OTS was exploring the relationship between OTS ROIs and target ROIs. In this study we focused on 3 target ROIs: ERC, hippocampus and the temporal MetaROI. We chose to examine ERC and hippocampus because these are among the earliest regions to accumulate tau, including in unimpaired older adults. Notably, with MK6240, unlike flortaucipir, quantification of hippocampal signal is feasible because MK6240 OTS is minimal in choroid plexus, which has been shown to contaminate hippocampal signal with flortaucipir. ERC and hippocampus were defined using FreeSurfer and the MetaROI was created by combining the following FreeSurfer ROIs: amygdala, ERC, fusiform gyrus and inferior and middle temporal gyri. The MetaROI is a summary measure of overall AD-specific tau burden (Jack et al., 2017; Ossenkoppele et al., 2021).

### 2.5. Imaging processing: OTS tertiles for visualizing the effect of OTS on target ROI SUVR

To assess the effect of meningeal OTS contamination in target ROIs we binned participants by OTS SUVR tertiles (Supplementary Fig. S1) in each OTS ROI and then plotted target ROI SUVRs by the corresponding OTS tertiles. Tertiles were used for better visualization of OTS effects, but all associations were also examined using continuous OTS data. Specifically, ERC SUVR was plotted by ERC OTS tertiles and the MetaROI SUVR was plotted by MetaROI OTS tertiles. Similarly, to explore the relationship between OTS adjacent to the cerebellum and spill-in to the reference region, we calculated inferior cerebellar gray matter SUVRs by intensity normalizing using unmasked Braak V/VI regions (Baker et al., 2017; Maass et al., 2017) where we do not expect substantial tau accumulation in this cohort of individuals without significant clinical impairment.

### 2.6. Image processing: Candidate processing approaches

To optimize our MK6240 processing pipeline, several approaches were evaluated. Target region SUVRs were calculated using an inferior cerebellar gray reference region (standard processing as originally developed for flortaucipir; (Baker et al., 2017; Maass et al., 2017)) or an eroded version (non-standard). To erode the inferior cerebellar grey matter region, we smoothed each binary native space MRI ROI image with an 8mm kernel and then thresholded at 0.8. This threshold was determined to best reduce OTS while retaining a large enough reference region to be stable and contiguous (Supplementary Fig. S2).

In addition, MK6240 scans were either unmasked (standard processing) or masked with the whole OTS mask described above (non-standard). The reverse-normalized whole OTS masks were allowed to overlap with (and thus remove) voxels in native space cortical regions defined by FreeSurfer (Supplementary Fig. S3). Reference region SUVR, however, was calculated in the whole ROI regardless of OTS mask overlap: this was to ensure reference region erosion could be performed in a standardized way and the effects of erosion evaluated separately. We also explored masking with a subject-specific brain mask defined using FreeSurfer (non-standard), which did not overlap with cortical or cerebellar voxels and present these results in the Supplementary Materials. In pipelines where we used a mask (either whole OTS or the brain mask) to remove meningeal OTS, the mask was applied prior to spatial smoothing and then an edge-preserving smoothing approach (non-standard) was used to avoid smoothing zero-value voxels from outside the brain into target regions and introducing resolution-dependent signal differences that differ across scanners. Edge preserving smoothing was achieved by smoothing the binary brain mask at the same time as the PET image. The resulting volume represents the weight that zero-value voxels outside the brain have on those inside the brain. By dividing the smoothed PET image by this smoothed mask, we removed the influence of zero-value voxels outside the brain. As with standard spatial smoothing, edge-preserving smoothing was applied using different kernels to achieve a common resolution of 6mm^3^ isotropic.

Finally, we applied PVC using a geometric transfer matrix approach (Rousset et al., 1998) with ROIs optimized for MK6240: FreeSurfer-segmented ROIs, inferior cerebellar gray, CSF, skull, meninges and subject-specific OTS regions were defined based on peak activity (Baker et al., 2017). In all this resulted in 7 distinct processing approaches that are listed in Table 1. We used the target ROIs described above (ERC, hippocampus and a temporal MetaROI) to assess the 7 processing approaches, including by measuring the effect size of the difference in MK6240 SUVR between A *β* status groups.

### 2.7. Statistical analysis

First, Pearson correlation was used to assess cross-correlations between OTS ROIs. Next, to determine if MK6240 meningeal OTS was related to biological variables we used linear regression for continuous variables (e.g., age) and two-sided t-tests for categorical variables (e.g., sex). Then, OTS ROI SUVRs were used to group participants into tertiles representing low, medium, and high OTS groups (Supplementary Fig. S1). Two-sided t-tests were used to compare target region MK6240 SUVR across OTS tertile groups. To assess the effect of non-standard processing approaches on target region SUVRs we subtracted target region SUVR using standard processing from the same region SUVR using one of the non-standard, novel approaches. This change in SUVR (ΔSUVR) was plotted against OTS in corresponding regions (e.g., ΔSUVR in ERC vs ERC OTS) and linear regression was used to examine associations. Finally, the relationships between continuous MK6240 target region SUVR with age, sex, A *β* and cognitive status were observed using two-sided t-tests for categorical variables and linear regression for continuous variables.

## 3. Results

### 3.1. Participants

Cohort characteristics are shown in Table 2. 481 POINTER Imaging participants with baseline MK6240, FBB and structural MRI scans were included. The present study cohort represents a subset (~50%) of the projected total POINTER Imaging sample. Females were slightly over-represented comprising 66% of the sample. Notably, the proportion of individuals from ethnic or racial underrepresented groups (URG) was 27%. To explore the effect of race on MK6240 binding, participants were binned into URG (non-white) or white racial categories. The participants in this study are community-dwelling, predominantly cognitively unimpaired older adults, but 22% had a global CDR score of 0.5 which indicates the possibility of mild cognitive impairment. Participants with significant cognitive impairment (TICSm <32 or CDR Sum of Boxes> 1.0) were screened out of the parent trial.

### 3.2. Greater meningeal OTS is unimodally distributed and related to younger age and female sex

Meningeal OTS was visualized by warping A *β*- participants’ scans to template space and averaging them together (Fig. 1A). In native space for each participant, we calculated SUVRs in each of the four OTS ROIs (Fig. 1B; for additional views of whole OTS mask see Supplementary Fig. S3) and plotted their distributions (Fig. 1C). For each OTS ROI, the distributions of SUVRs were unimodal with a long right-sided tail. OTS severity occurred on a spectrum and there was no suggestion of OTS ‘groups’ or categorical incidence of meningeal OTS. Pearson correlations between OTS ROIs were high overall (all p < 0.001) with slightly lower associations with ERC OTS compared to cross-correlations with whole OTS, MetaROI OTS and inferior cerebellar OTS (Fig. 1D).

**Fig. 2.**
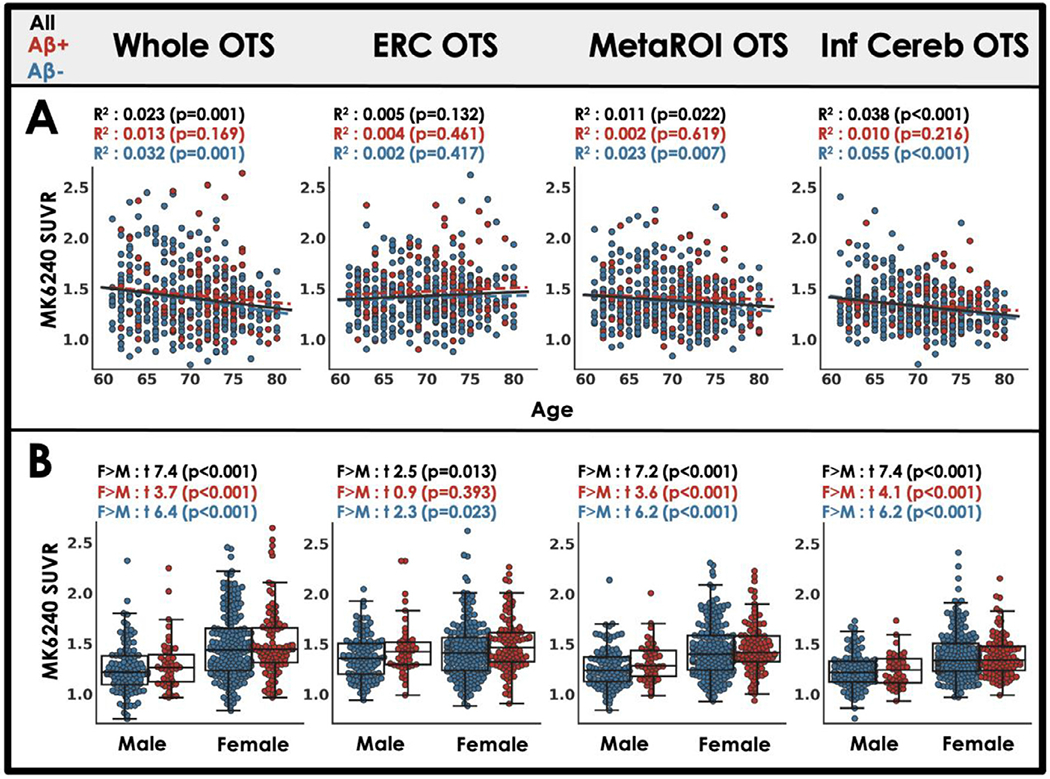
OTS SUVR relationships to age and sex (A) Bivariate associations from least squares regression between non-adjusted age and OTS ROI SUVR are shown by the coefficient of discrimination and statistical significance (n=481). (B) The effect of sex on OTS ROI SUVR is tested with Student’s t-test. Statistical tests are repeated for A *β* negative (blue) and amyloid positive (red) subsets of the whole cohort.

Next, we examined relationships between OTS and biological variables including age, sex, and race. Across the whole sample, there were negative correlations between OTS and age except in the ERC OTS ROI (Fig. 2A). When examining the relationship between OTS and sex, we observed a robust effect of sex such that females had greater OTS than males in the whole sample (see Supplementary Fig. S4 for mean SUVR images by sex) as well as in A *β* −/ + subgroups, with the exception of A *β*+ participants for ERC OTS (Fig. 2B). There was no significant effect of race on OTS in any OTS ROI (Supplementary Table S1).

### 3.3. OTS in the meninges is related to target regions

After quantifying OTS across several meningeal ROIs, we wanted to demonstrate spill-in that occurs between OTS ROIs and target regions. To explore this, we first created groups based on OTS SUVR tertiles in each OTS ROI and then plotted target ROI SUVRs by the corresponding OTS tertiles. Using our standard MRI-guided processing, we observed that target region and reference region SUVRs track with adjacent OTS tertiles (Supplementary Fig. S5). We also observed this when using OTS as a continuous variable (Supplementary Table S2). These patterns are likely caused by bidirectional partial volume effects (OTS ROI ← → target ROI). OTS contamination can be demonstrated at the single participant level, where we showed that SUVRs in target regions adjacent to meningeal OTS continue to decrease as the target region is eroded farther away from the meninges (Supplementary Fig. S6).

### 3.4. Reducing meningeal OTS contamination in cerebellum and target regions has opposing effects

**Fig. 3.**
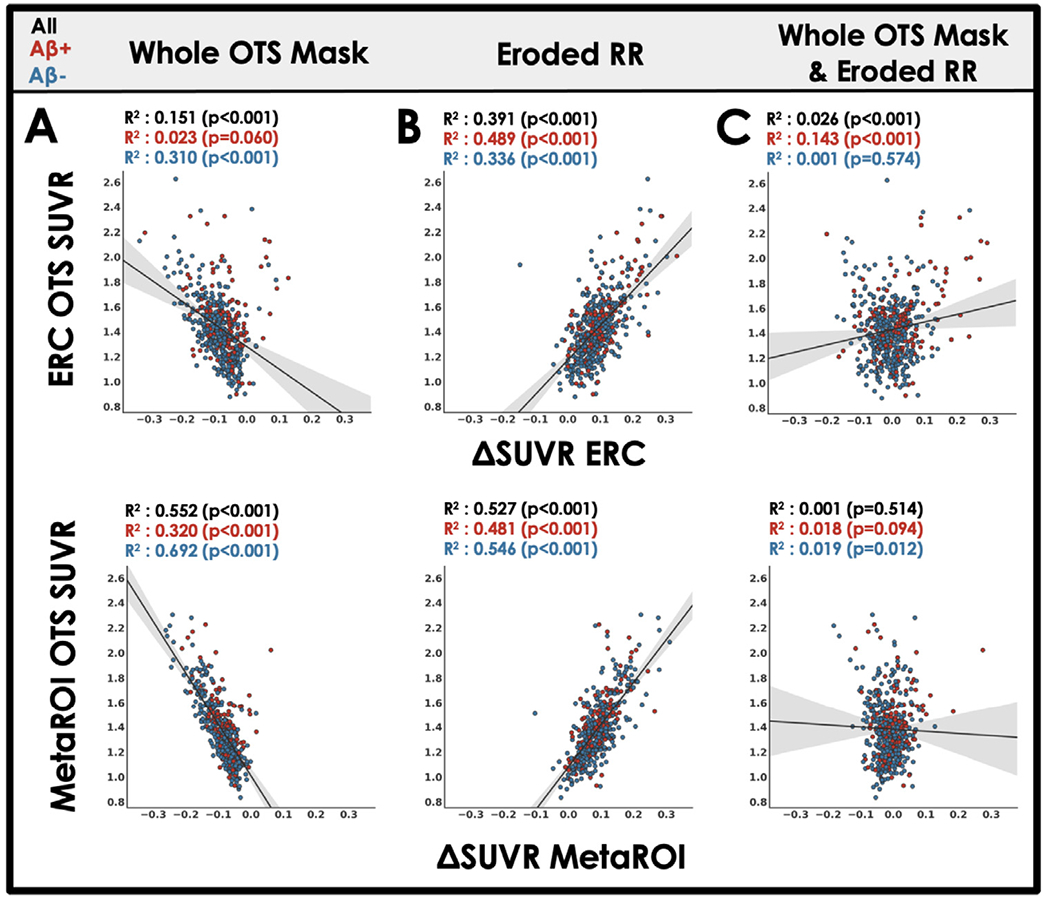
The effect of processing steps to mitigate OTS on target region SUVR. Scatter-plot and least squares regression of MK6240 OTS SUVR bordering regions of interest (Fig. 1B) against the change (Δ) in target region SUVR when applying processing steps (A-C) intended to mitigate OTS spill-in (Table 1). Results are described by the coefficient of determination and statistical significance. Statistical tests are repeated for A *β* negative (blue) and A *β* positive (red) subsets of the whole cohort.

After we observed evidence that meningeal OTS with MK6240 spills into target ROIs, (such as ERC, the MetaROI) and the reference region often used for this tracer (inferior cerebellar gray matter) we devised approaches to remove meningeal OTS from cortical regions and from the reference region. The cohort-specific ‘whole OTS’ mask, which was created to include all of the meninges, was used to mask MK6240 scans to remove OTS before edge-preserving spatial smoothing. Compared to our standard MRI-guided processing, masking with the whole OTS mask yields lower SUVRs (negative ΔSUVR relative to our standard processing approach) in target regions, presumably due to successful reduction of meningeal OTS spill-in in target regions (Fig. 3A). In contrast, eroding the reference region results in higher SUVRs in target regions, presumably due to successful reduction of meningeal OTS from the reference region (Fig. 3B). While it is intuitive that removing OTS from target and reference regions would result in more accurate quantification, we found that these steps had opposing effects that essentially canceled each other out. Thus, for the whole sample using our hypothetically optimized pipeline where the meninges were removed with the whole OTS mask and the reference region was eroded, the ERC ΔSUVR was weakly correlated to ERC OTS and there was no significant correlation between MetaROI ΔSUVR and MetaROI OTS (Fig. 3C). This indicates that the changes introduced by our modifications in the optimized pipeline (ΔUVR) are not related to OTS and that optimized processing SUVRs are highly correlated with standard processing SUVRs (Supplementary Fig. S7).

### 3.5. OTS-associated trends in target regions largely remained regardless of processing approach

**Fig. 4.**
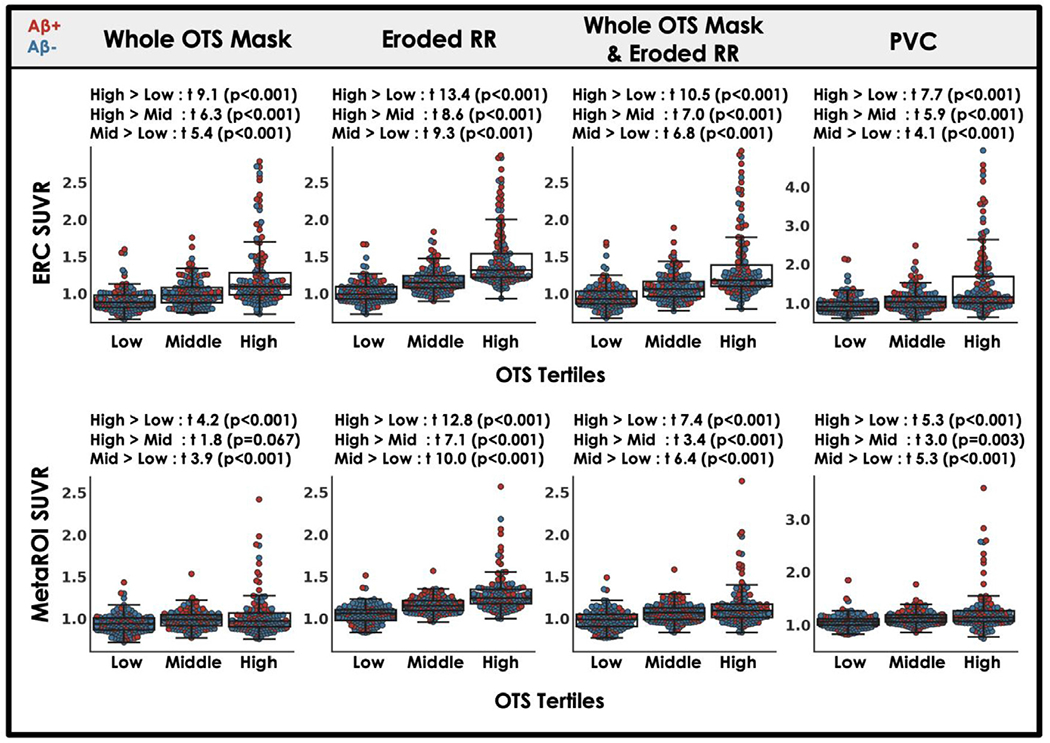
Candidate processing approaches do not eliminate the effect of OTS SUVR tertile on target region quantification. [18F]MK6240 OTS ROI (see Fig. 1B) SUVR was used to divide the cohort into tertiles: low, middle, and high OTS. SUVR values within target regions are plotted for each processing approach (Table 1) by OTS tertile. Student’s t-test describes the difference target region SUVR between OTS groups. Individual points are labeled by A *β* status: positive (red) and negative (blue)

We observed that OTS was related to target region SUVRs using our standard MRI-guided processing approach (Supplementary Fig. S5). This relationship between OTS and target region SUVR remained in each of our alternative processing approaches (Fig. 4). However, applying the whole OTS mask without eroding the reference region appeared to diminish, but not eliminate, the stepwise increase in target regions SUVRs by OTS tertiles. In contrast, eroding the reference region without applying a global meningeal mask exaggerated the OTS effects in target regions. OTS tertile results for two additional approaches, brainmask and brainmask plus eroded reference region (Table 1), are reported in Supplementary Table S3.

### 3.6. Each processing approach shows similar tau relationships with age, A *β* and memory

**Fig. 5.**
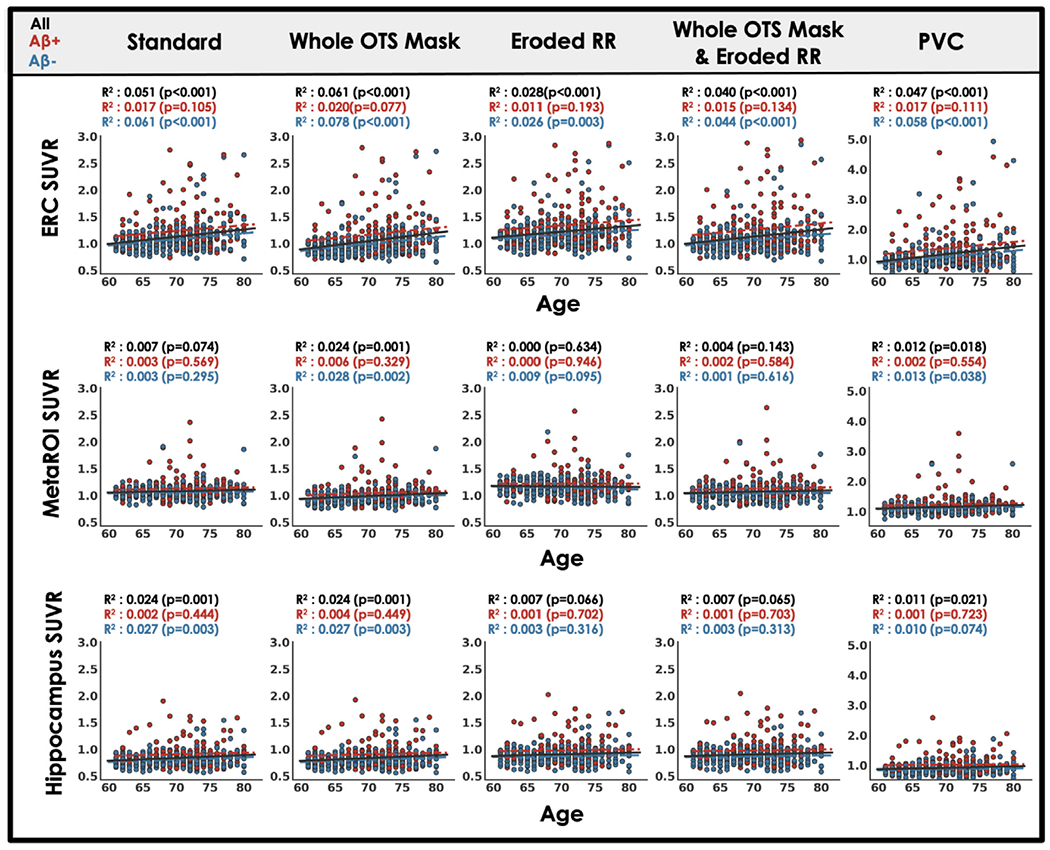
Target region MK6240 SUVR relationships to age across processing approaches. Scatterplot and least squares regression of MK6240 SUVR in three target regions against age for five processing approaches (Table 1; see Supplementary Table S4 for results with additional candidate approaches). Results are described by the coefficient of determination and statistical significance. Statistical tests are repeated for A *β* negative (blue) and A *β* positive (red) subsets of the whole cohort.

**Fig. 6.**
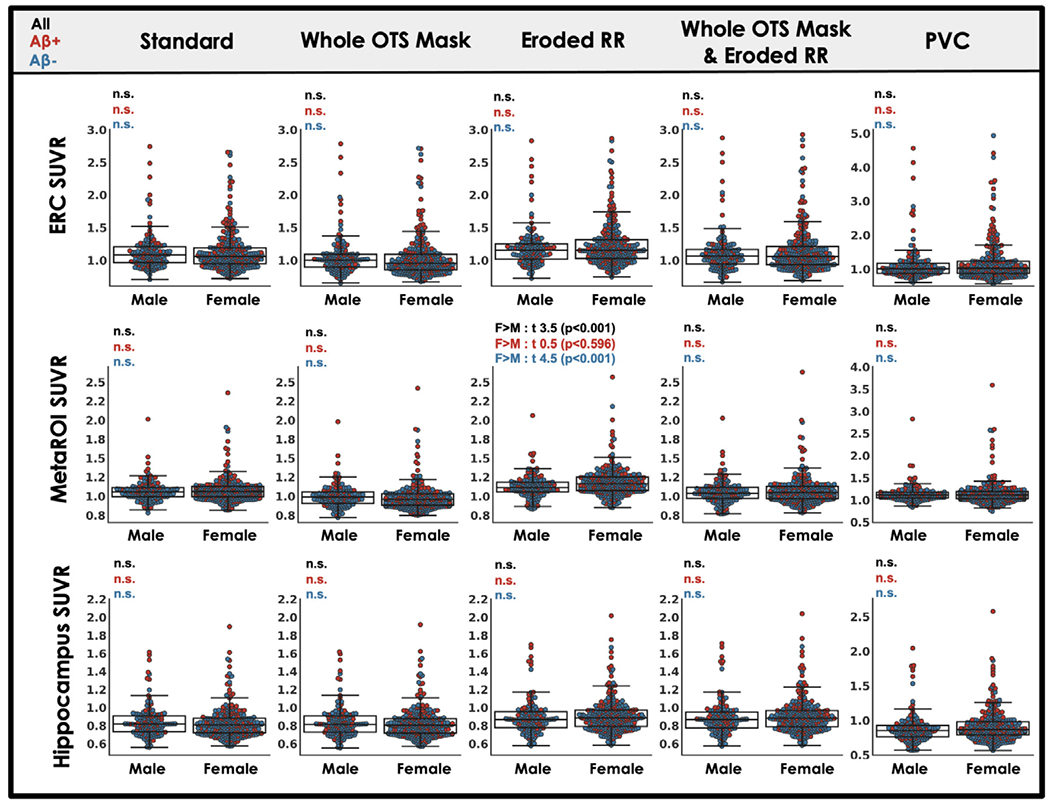
Target region MK6240 SUVR relationships to sex across processing approaches. MK6240 SUVR in three target regions is plotted by sex for five processing approaches (Table 1; see Supplementary Table S4 for results with additional candidate approaches). Student’s t-test describes the difference between male and female groups. Statistical tests are repeated for A *β* negative (blue) and A *β* positive (red) subsets of the whole cohort.

**Fig. 7.**
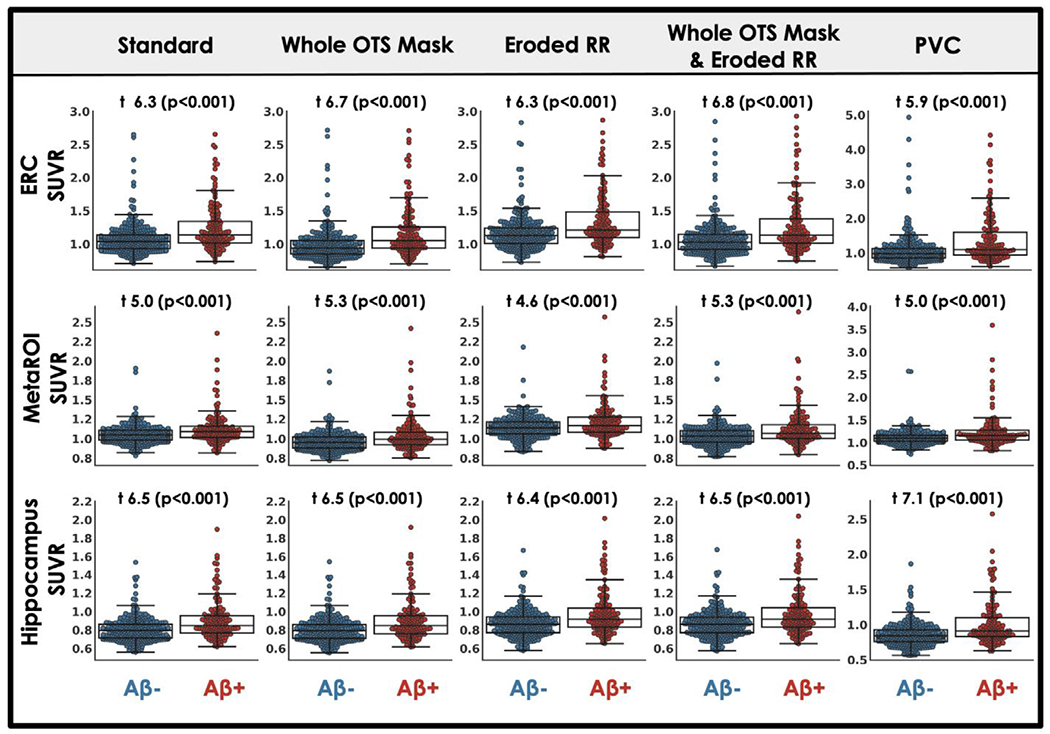
Target region MK6240 SUVR relationships to A *β* status across processing approaches. MK6240 SUVR in three target regions is plotted by A *β* status for five processing approaches (Table 1; see Supplementary Table S4 for results with additional candidate approaches). Student’s t-test describes the difference between A *β* negative (A *β*−) and A *β* positive (A *β*+) groups.

**Fig. 8.**
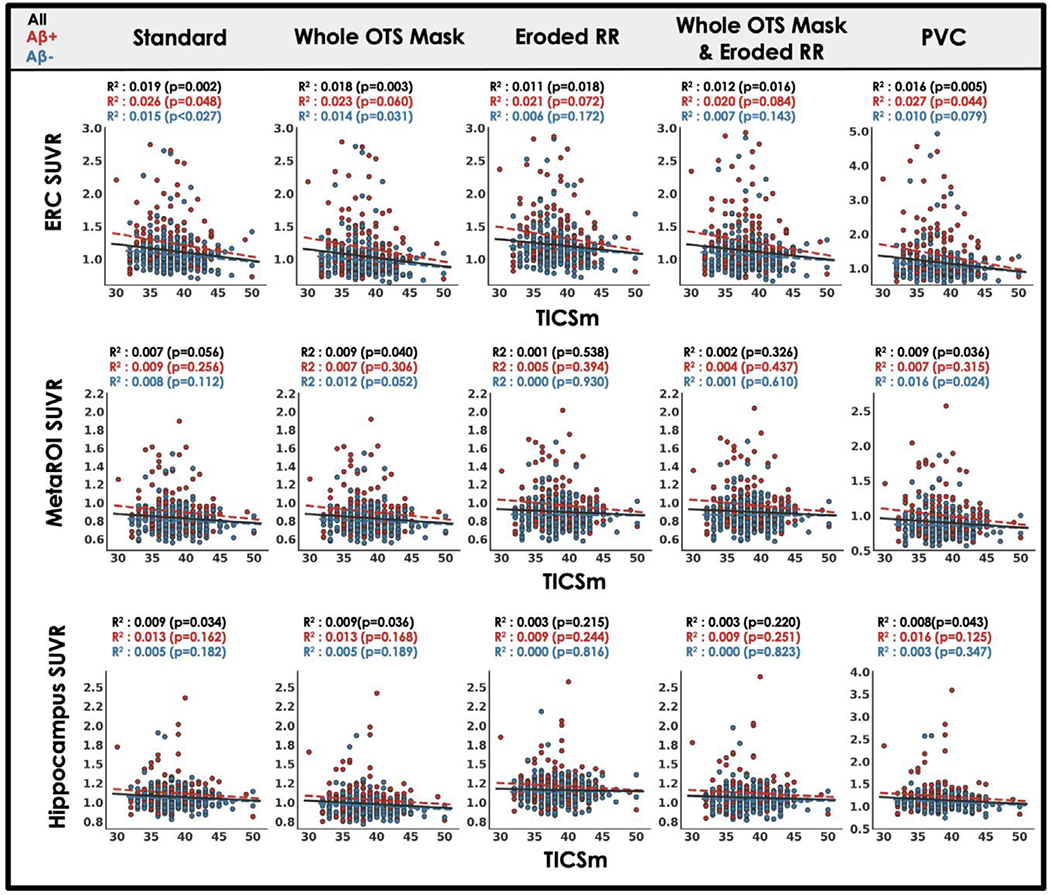
Target region MK6240 SUVR relationships to general cognition (TICSm) across processing approaches. Scatterplot and least squares regression of MK6240 SUVR in three target regions against TICSm total for five processing approaches (Table 1; see Supplementary Table S4 for results with additional candidate approaches). Results are described by the coefficient of determination and statistical significance. Statistical tests are repeated for A *β* negative (blue) and A *β* positive (red) subsets of the whole cohort.

Across processing approaches, including our standard processing, MK6240 SUVR measured in target regions showed expected relationships with age, A *β* and cognition. Relationships with sex and race (URG vs. white) were also explored. For each processing approach, ERC MK6240 SUVR was positively correlated with age while the other target regions, hippocampus and MetaROI, generally showed no association with age (Fig. 5). We observed no effect of sex on MK6240 SUVR in any target region for any processing approach except for the MetaROI when only the reference region was eroded (Fig. 6). We believe this effect is driven by the enhanced influence of OTS on target regions that occurs when the reference region is eroded (Fig. 4) and the robust F > M sex effect in meningeal OTS with this tracer (Fig. 2B). Regardless of processing approach there is a significant effect of A *β* such that A *β*+ participants have greater MK6240 SUVR in target regions compared to A *β*− participants (Fig. 7). A *β*−/ + effect size comparison reveals similar results across candidate approaches. Finally, there was a significant negative association between MK6240 SUVR in target regions and general cognitive status (TICSm) which was present for each tau-PET processing approach, with slightly stronger associations when using PVC (Fig. 8). Using standard processing, there was no significant effect of race in ERC and hippocampus and a borderline significant effect (p=0.048) of greater MK6240 binding in the MetaROI in white participants (Supplementary Table S1). There were no significant effects of race on target regions using alternative processing approaches. Results of age, sex, A *β* and TICSm relationships with target region MK6240 SUVR for two additional processing approaches, brainmask and brainmask plus eroded reference region (Table 1), are reported in Supplementary Table S4.

## 4. Discussion

In the present study, we compared strategies for mitigating the influence of meningeal OTS on quantification of cross-sectional MK6240 scans in nearly 500 older adults without significant cognitive impairment. OTS was visually striking, especially in individuals with overall low tau burden, and variability in this signal was roughly normally distributed. We assessed the severity and distribution of OTS, related OTS to basic demographic and biomarker data and assessed various processing approaches to reduce OTS and optimize regional quantification. We demonstrated that meningeal OTS is associated with signal in cortical regions in MK6240 scans. This is theoretically especially problematic in older adults with lower overall tau burden because the influence of this signal on target region SUVR estimation is relatively larger. OTS also was higher in younger individuals and in females. We employed several strategies to remove OTS from target regions but found that, in general, attempts to remove OTS did not improve our ability to detect previously-reported associations with increased A *β*, older age, and female sex, compared to standard processing. Because OTS neighboring target and reference regions was correlated within participant (Fig. 1) OTS contamination largely canceled out, resulting in SUVRs that were similar to those generated after more involved processing methods that specifically aimed to reduce effects of OTS.

While developing our MK6240 processing pipeline we examined 6 alternative approaches to our standard MRI-guided PET processing. The rationale for each of these approaches is briefly described in Table 1. Previous studies with MK6240 have implemented some of these approaches including eroding the reference region (Betthauser et al., 2019) or masking out the meninges before smoothing (Krishnadas et al., 2022; Pascoal et al., 2021, 2020). In addition, a recent study examined the effects of PVC in MK6240 and found that meningeal OTS spill-in effects (high OTS vs low OTS comparisons) could be significantly reduced using PVC strategies (Mertens et al., 2022), a result we did not replicate (Fig. 4). To our knowledge, the present study is the first to put these various methods head-to-head to assess whether tau associations (e.g., with age, sex or A *β*) can be improved by reducing meningeal OTS spill-in to cortical regions.

Our work suggests that meningeal OTS in MK6240 is unimodally distributed, which makes quantifying the prevalence of OTS challenging as it is a continuous phenomenon. Similarly, given the unimodal distribution, deriving a cut-off to exclude participants with high OTS is not a viable approach. We therefore reasoned that specific preprocessing strategies applied to all scans would be the best approach to reducing OTS in our dataset. However, our comparison of processing approaches showed that correcting for OTS is not straightforward as methods for alleviating the effects of OTS in target regions and the reference region have similar, opposing effects on SUVR. The opposing effects of eroding the reference region and applying a meningeal mask to cortical regions therefore resulted in SUVRs that were highly correlated with our standard processing. By extension, if attempts are made to remove OTS from only target regions or only the reference region this will introduce changes in target region SUVRs that are driven by OTS in the regions where it has not been reduced. For example, eroding the reference region without also masking out OTS from target regions results in higher SUVRs in target regions driven by adjacent OTS and should be avoided. We see this in Fig. 6 where we observe a tau by sex effect in the MetaROI when the reference region is eroded but target regions are not. This finding is misleading and likely driven by the robust F > M sex effect in meningeal OTS. Relatedly, while the focus of this study was effects of OTS spill-in to target regions, it is also true that on-target signal (i.e., tracer binding to tau pathology) spills-in to OTS ROIs. In fact, relatively lower correlations with ERC OTS across the four ROIs (Fig. 1D) could be the result of actual ERC tau binding spilling outside the brain.

We explored MK6240 tracer binding in target ROIs, regions of early tau accumulation, and regions of OTS with demographic variables of interest including age, sex, and race. We observed opposing effects of age in target ROIs (positive correlations with age) and OTS ROIs (negative correlations with age). This suggests that the tau and age association we observe, especially in ERC, is not driven by OTS which would actually weaken the expected positive association. A separate study reported negative associations between age and meningeal OTS in two other tau-PET tracers, flortaucipir and RO948, but not in MK6240 (Smith et al., 2021). However, their MK6240 sample was 62% smaller so the lack of effect may have been related to statistical power.

In contrast, despite our relatively large cohort, we did not observe an effect of sex in our target ROIs. This result is somewhat surprising given previously published reports of elevated tau measured in neuropathology samples (Barnes et al., 2005; Spina et al., 2021), as well as PET or CSF tau markers (Buckley et al., 2022, 2019; Hohman et al., 2018) suggesting that women have greater tau pathology than men, even in unimpaired older adults. One possible explanation for this difference is that these reported sex effects were often only in participants who carry the AD risk gene APOE*ε*4, data that are currently not available for the POINTER participants. There is also some evidence that female A *β*+ individuals have greater tau than their male counterparts, but we did not replicate this finding with the current POINTER Imaging dataset. Another reason we may not observe the sex effect in tau pathology is unknown interactions between sex and cardiovascular risk factors that are enriched in the POINTER Imaging cohort. As the POINTER Imaging cohort grows, (the present study includes ~50% of targeted enrollment) there may be sufficient power to detect a sex effect but we would expect the effect size to be modest. We did, however, observe a strong effect of sex on OTS with females showing higher magnitude OTS, which is consistent with a previous report showing higher meningeal OTS in women in three different tau-PET tracers including MK6240 (Smith et al., 2021). Importantly, our OTS comparisons were done using native space OTS ROIs for ERC, MetaROI and inferior cerebellar gray to avoid a possible sex effect driven by warping biases due to head size differences between men and women but this could still play a role given the reverse normalization of the whole OTS mask. The reason for higher meningeal OTS in women is unknown. One factor that may contribute is the relatively higher incidence of hyperostosis frontalis interna in women. This benign thickening of the frontal bone is relatively rare but appears to coincide with very high MK6240 OTS. Other possible explanations related to known sex differences in hormones and metabolism are speculative.

This study had unique strengths as well as limitations. Strengths included the large dataset with nearly 500 participants, the use of native space anatomical information for quantification, and the relatively large proportion of URG individuals who have been historically underrepresented in research including in neuroimaging datasets. We did not observe any effect of race on OTS or target region SUVRs. Our simplistic approach testing race associations (URG vs white individuals) may hide patterns related to a single racial or ethnic group, which will be better addressed in the full POINTER Imaging sample. Limitations include relatively low tau pathology in our cohort of at-risk, unimpaired older adults that restricted our ability to assess effects of OTS in the context of higher tau accumulation. We focused on regions of early tau accumulation (ERC, MetaROI) for evaluating pipeline performance. Thus, it is possible that the optimal processing for other brain regions less prone to meningeal OTS may differ. In addition, the data in this study were cross-sectional so we were unable to compare longitudinal changes in OTS to changes in target regions. Future work will assess optimal methods for processing longitudinal MK6240 data. Another constraint on this work was the limited cognitive data currently available for analyses. POINTER trial participants undergo sensitive and domain-specific cognitive testing and these scores will become available as the POINTER trial releases data. Because detection of early tau associated with episodic memory cognitive decline is of critical importance, further work is needed to as-sess the sensitivity of the MK6240 tracer to memory-associated early tau.

## 5. Conclusions

The tau-PET tracer MK6240 suffers from OTS in the meninges and this signal contaminates cortical regions and the cerebellum. In addition to our standard MRI-guided PET processing, we examined 6 alternative approaches designed to reduce the effect of OTS on target region quantification. Our efforts revealed that none of the alternative processing approaches we tested improved effect sizes for key associations between MK6240 target region SUVR and age, sex, and A *β*. This is reassuring as it suggests that while MK6240 OTS contamination certainly introduces noise to the data, the tau signal is not obfuscated by this noise.

## Supplementary Material

Supplementary Material

## Figures and Tables

**Table 1:** MK6240 Candidate Processing Approaches

	Summary	Rationale
**Standard MRI-Guided Processing**	PET frames averaged and smoothed; PET scan co-registered to MRI; FS native space regions used to calculate SUVRs	Default approach validated for other tau-PET tracers and used to compare to candidate MK6240-specific approaches.
**‘Whole OTS’ Mask**	Before smoothing, apply whole OTS mask to PET image; results in some erosion of cortical regions; use edge-preserving smoothing to achieve common resolution	Masks out meninges and voxels from target regions predicted to be unreliable based on the reverse-normalized whole OTS mask.
**Eroded RR**	Erode the edge of the inferior cerebellar GM mask by smoothing the original ROI and thresholding at 0.8	Removes RR voxels that are likely to be contaminated by meningeal OTS.
**‘Whole OTS’ Mask & Eroded RR**	Combination of whole OTS mask and eroded RR approaches.	A hypothetically optimized approach that reduced OTS in both target regions and the reference region.
**GTM PVC**	PVC by GTM; ROIs: all FS regions, tissue masks C3 and C4+5 from SPM12, inferior cerebellar GM and subject-specific extracortical hot-spots.	Adjusting ROI intensity based on assumptions of partial volume effects and image resolution.
**Brainmask** ^†^	Before smoothing, apply FS-derived brainmask to PET image; masks out meninges but does not remove any voxels from FS ROIs	To contrast to the use of the whole OTS mask which removed meninges and cortical voxels likely to be contaminated by OTS spill-in
**Brainmask & Eroded RR** ^†^	Combination of FS brainmask and eroded RR approaches.	To contrast to the whole OTS mask & eroded RR.

**Abbreviations:** MRI=magnetic resonance imaging; PET=positron emission tomography; NA=not applicable; FS=FreeSurfer; ROIs=regions of interest; OTS=off-target signal; RR=reference region; GM=gray matter; GTM=geometric transfer matrix; PVC=partial volume correction

† Results described/shown in the Supplementary Materials

**Table 2:** Cohort Characteristics

	POINTER Imaging (n=481)
**Age** (yrs)	70.0 [5.0]
**Sex** (M / F)	162 / 319 (66%)
**Race** (white / URG [non-white])	349 / 132 (27%)
**CDR** (0 / 0.5)	376 / 105 (22%)
**TICSm**	38.4 [3.2]
**MK6240 ERC**	1.12 [0.29]
**MK6240 MetaROI**	1.07 [0.15]
**A *β* (FBB) Status** (− / +)	328 / 153 (31%)
**FBB Summary** SUVR / CL	1.08 [0.17] / 17.7 [26.5]

Reporting mean [standard deviation] for continuous variables.

**Abbreviations:** yrs=years; M=male; F=female; CDR=clinical dementia rating; TICSm=Modified Telephone Interview for Cognitive Status; ERC=entorhinal cortex; MetaROI = femporal meta region of interest; A *β*=beta-amyloid; FBB=florbetaben; SUVR=standardized uptake value ratio; CL=centiloid

## Data Availability

Data will be made available on request.
