# Supplementary material for: Optimizing quantification of MK6240 tau PET in unimpaired older adults
Source: Neuroimage. Author manuscript; Available in PMC 2023 Feb 24. (PMC9957642; doi:10.1016/j.neuroimage.2022.119761)
Supplement: Supplementary Material [file NIHMS1872566-supplement-Supplementary_Material.docx]

***Supplementary Materials***

**Tables**

**Supplementary Table S1**

**Race (URG > white) Differences in MK6240 SUVR**

|  | **t** | ***p*** |
| --- | --- | --- |
| **Whole OTS** | -0.605 | 0.545 |
| **ERC OTS** | -1.779 | 0.076 |
| **MetaROI OTS** | -1.203 | 0.230 |
| **Inferior Cerebellum OTS** | -0.353 | 0.724 |
| **ERC** | -1.772 | 0.077 |
| **MetaROI** | -1.982 | *0.048* |
| **Hippocampus** | -1.523 | 0.128 |

**Abbreviations:** URG=underrepresented groups; SUVR=standardized uptake value ratio; OTS=off-target signal; ERC=entorhinal cortex

**Supplementary Table S2**

**Continuous MK6240 OTS and Target Region SUVR Regression**

|  | **R^2^** | ***p*** |
| --- | --- | --- |
| **ERC** | 0.422 | *<0.001* |
| **MetaROI** | 0.210 | *<0.001* |
| **Inf Cereb GM** | 0.136 | *<0.001* |

**Abbreviations:** OTS=off-target signal; SUVR=standardized uptake value ratio; ERC=entorhinal cortex; Inf Cereb GM=inferior cerebellar gray matter

**Supplementary Table S3**

**Additional Processing Approaches: MK6240 Target SUVR by OTS Tertiles**

| **ROI** |  | **Brainmask** | | **Brainmask & Eroded RR** | |
| --- | --- | --- | --- | --- | --- |
|  |  | **t** | ***p*** | **t** | ***p*** |
| **ERC**  **(by ERC OTS tertile)** | High> Low | 10.711 | <0.001 | 12.273 | <0.001 |
|  | High>Mid | 7.245 | <0.001 | 8.114 | <0.001 |
|  | Mid>Low | 6.498 | <0.001 | 8.002 | <0.001 |
| **MetaROI**  **(by MetaROI OTS tertile)** | High> Low | 7.332 | <0.001 | 11.123 | <0.001 |
|  | High>Mid | 3.645 | <0.001 | 5.859 | <0.001 |
|  | Mid>Low | 6.451 | <0.001 | 9.152 | <0.001 |

**Abbreviations:** SUVR=standardized uptake value ratio; ERC=entorhinal cortex; OTS=off-target signal; RR=reference region

**Supplementary Table S4**

**Additional Processing Approaches: MK6240 Target SUVR with age, sex, Aβ and TICSm**

| **Analysis** | | **ROI** | **Group** | **Brainmask** | | **Brainmask & Eroded RR** | |
| --- | --- | --- | --- | --- | --- | --- | --- |
|  |  |  |  | **R^2^** | ***p*** | **R^2^** | ***p*** |
| **Age** | | **ERC** | All | 0.055 | <0.001 | 0.033 | <0.001 |
|  |  |  | Aβ+ | 0.018 | 0.101 | 0.012 | 0.179 |
|  |  |  | Aβ- | 0.068 | <0.001 | 0.034 | 0.001 |
|  |  | **MetaROI** | All | 0.009 | 0.035 | 0.000 | 0.935 |
|  |  |  | Aβ+ | 0.002 | 0.540 | 0.000 | 0.897 |
|  |  |  | Aβ- | 0.007 | 0.145 | 0.005 | 0.211 |
|  |  | **Hippocampus** | All | 0.024 | 0.001 | 0.007 | 0.064 |
|  |  |  | Aβ+ | 0.004 | 0.449 | 0.001 | 0.703 |
|  |  |  | Aβ- | 0.027 | 0.003 | 0.003 | 0.312 |
|  |  |  |  | **t** | **p** | **t** | **p** |
| **Sex (F>M)** | | **ERC** | All | -0.180 | 0.857 | 1.179 | 0.239 |
|  |  |  | Aβ+ | -0.319 | 0.750 | 0.279 | 0.781 |
|  |  |  | Aβ- | -0.436 | 0.663 | 1.034 | 0.302 |
|  |  | **MetaROI** | All | 0.496 | 0.620 | 2.954 | 0.003 |
|  |  |  | Aβ+ | -0.662 | 0.509 | 0.331 | 0.741 |
|  |  |  | Aβ- | 1.182 | 0.238 | 3.877 | <0.001 |
|  |  | **Hippocampus** | All | -0.791 | 0.429 | 1.039 | 0.299 |
|  |  |  | Aβ+ | -1.225 | 0.223 | -0.469 | 0.640 |
|  |  |  | Aβ- | -0.395 | 0.693 | 1.681 | 0.094 |
|  |  |  |  | **t** | **p** | **t** | **p** |
| **Aβ Status (Aβ+ > Aβ-)** | | **ERC** | All | 6.479 | <0.001 | 6.490 | <0.001 |
|  |  | **MetaROI** | All | 5.158 | <0.001 | 4.845 | <0.001 |
|  |  | **Hippocampus** | All | 6.533 | <0.001 | 6.485 | <0.001 |
|  |  |  |  | **R^2^** | **p** | **R^2^** | **p** |
| **TICSm** | | **ERC** | All | 0.018 | 0.003 | 0.011 | 0.018 |
|  |  |  | Aβ+ | 0.024 | 0.054 | 0.020 | 0.079 |
|  |  |  | Aβ- | 0.014 | 0.030 | 0.006 | 0.166 |
|  |  | **MetaROI** | All | 0.007 | 0.061 | 0.001 | 0.525 |
|  |  |  | Aβ+ | 0.006 | 0.323 | 0.003 | 0.476 |
|  |  |  | Aβ- | 0.009 | 0.090 | 0.000 | 0.949 |
|  |  | **Hippocampus** | All | 0.009 | 0.036 | 0.003 | 0.219 |
|  |  |  | Aβ+ | 0.013 | 0.168 | 0.009 | 0.251 |
|  |  |  | Aβ- | 0.005 | 0.189 | 0.000 | 0.822 |

**Abbreviations:** SUVR=standardized uptake value ratio; Aβ=beta-amyloid; TICSm=Modified Telephone Interview for Cognitive Status; ROI=region of interest; RR=reference region; ERC=entorhinal cortex; F=female; M=male

**Figures**

**Supplementary Figure S1**

**
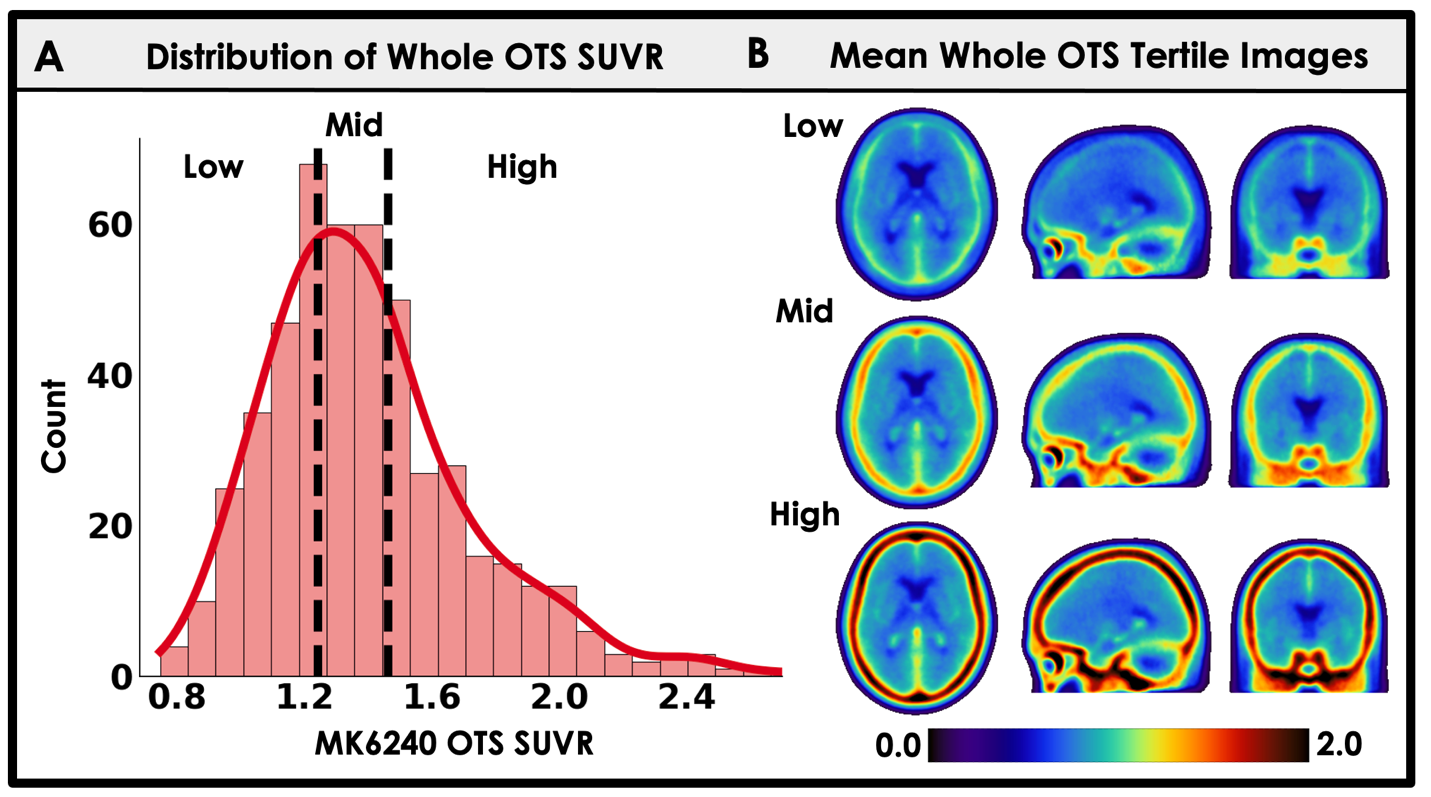
**

**Figure S1: OTS Tertiles.** For each OTS ROI the SUVR frequency distribution was plotted and divided into tertiles (dotted lines) with an equal number of participants in each. For example, the distribution shown in (A) is for the whole OTS mask (see Fig. 1B and Fig. S3A). (B) For visualization, average template space images of each tertile group (low, medium and high) based on the whole OTS mask are shown. Colorbar represents mean SUVR.

**Supplementary Figure S2**

**
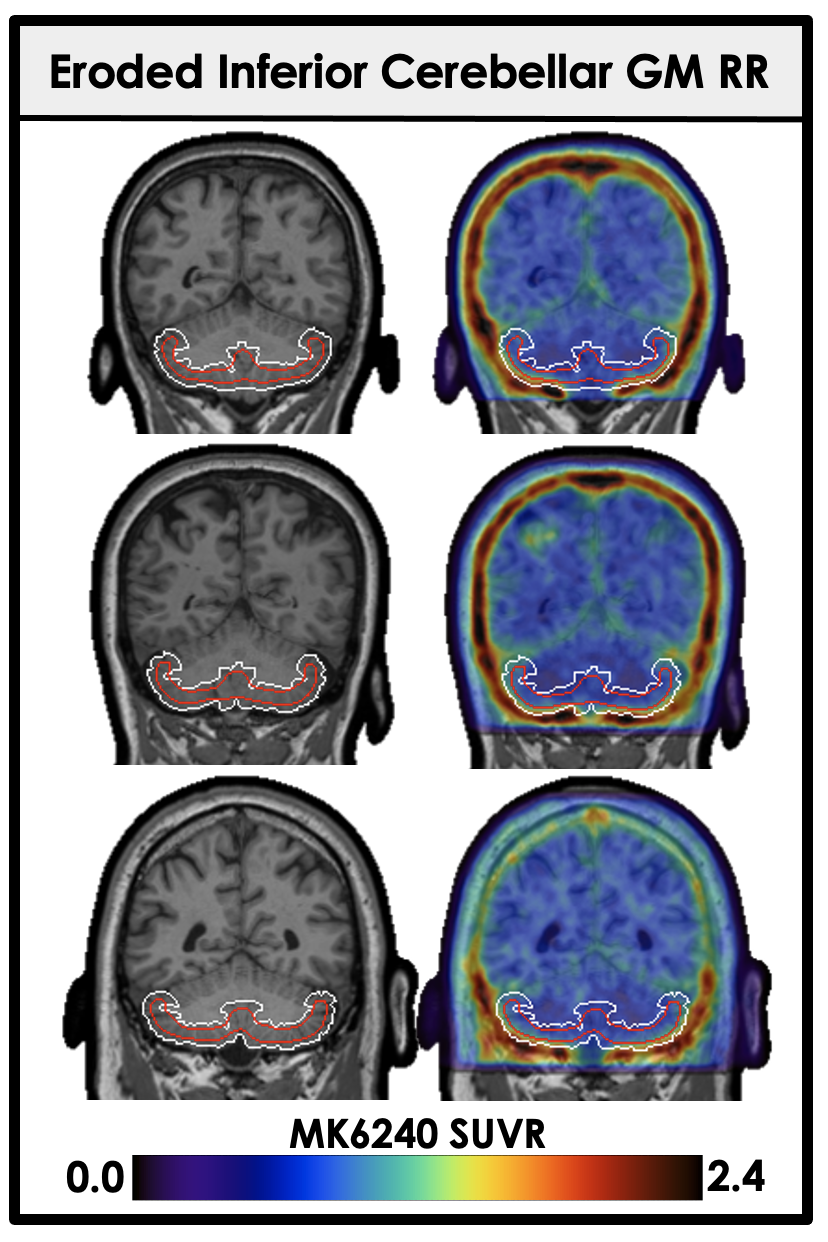
**

**Figure S2: Eroding the inferior cerebellum gray matter.** Three participant examples of uneroded (white outline) and eroded (red outline) inferior cerebellum reference regions in native space overlaid on MRI alone (left) and with an additional MK6240 PET overlay (right). Colorbar represents SUVR.

**Supplementary Figure S3**

**
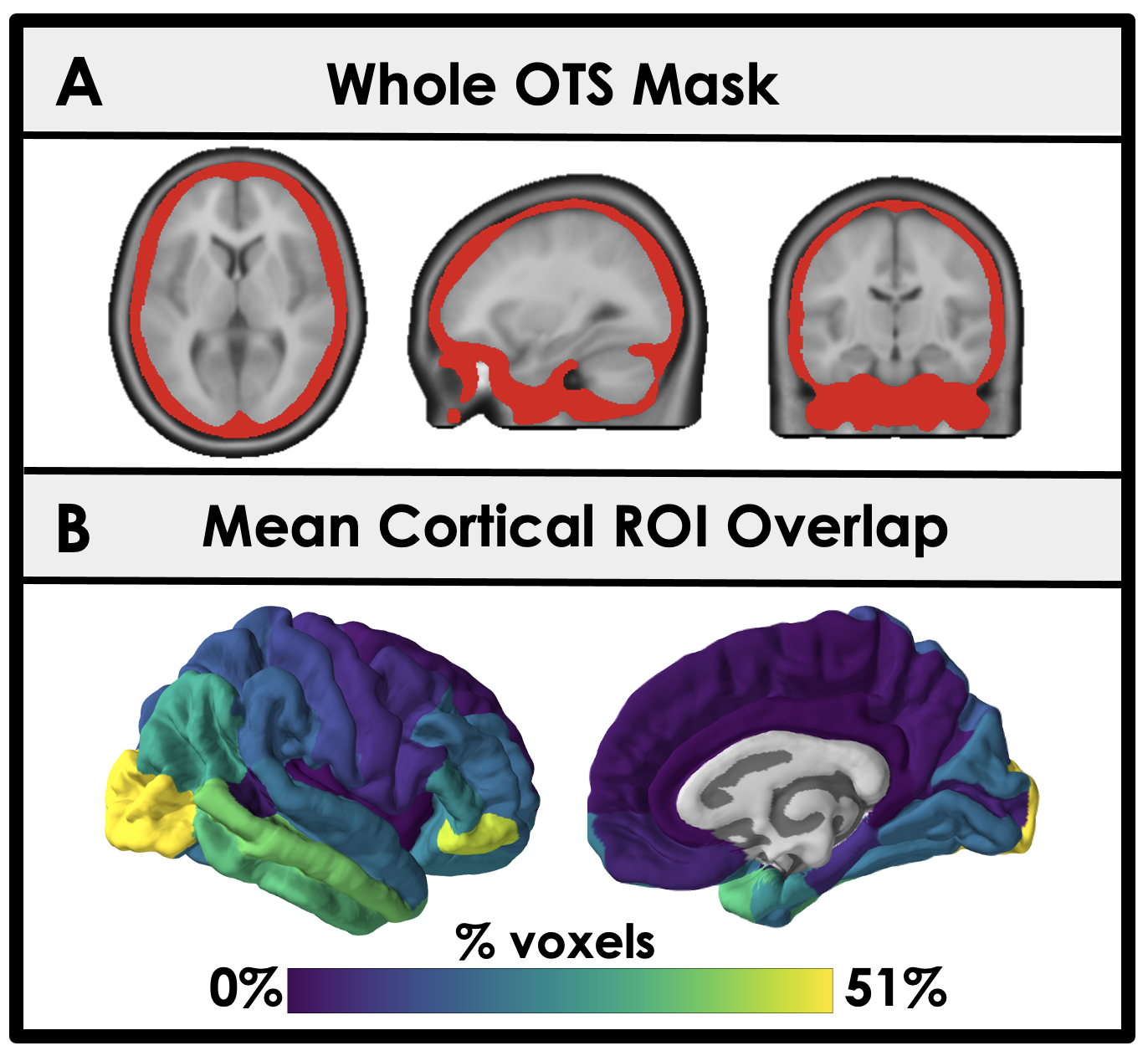
**

**Figure S3: Reverse normalization of the whole OTS mask results in overlap with cortical voxels.** (A) Axial, sagittal and coronal views of the whole OTS mask. (B) The across-participants mean percent of voxels in each FS cortical ROI that overlapped with reverse-normalized whole OTS mask.

**Supplementary Figure S4**


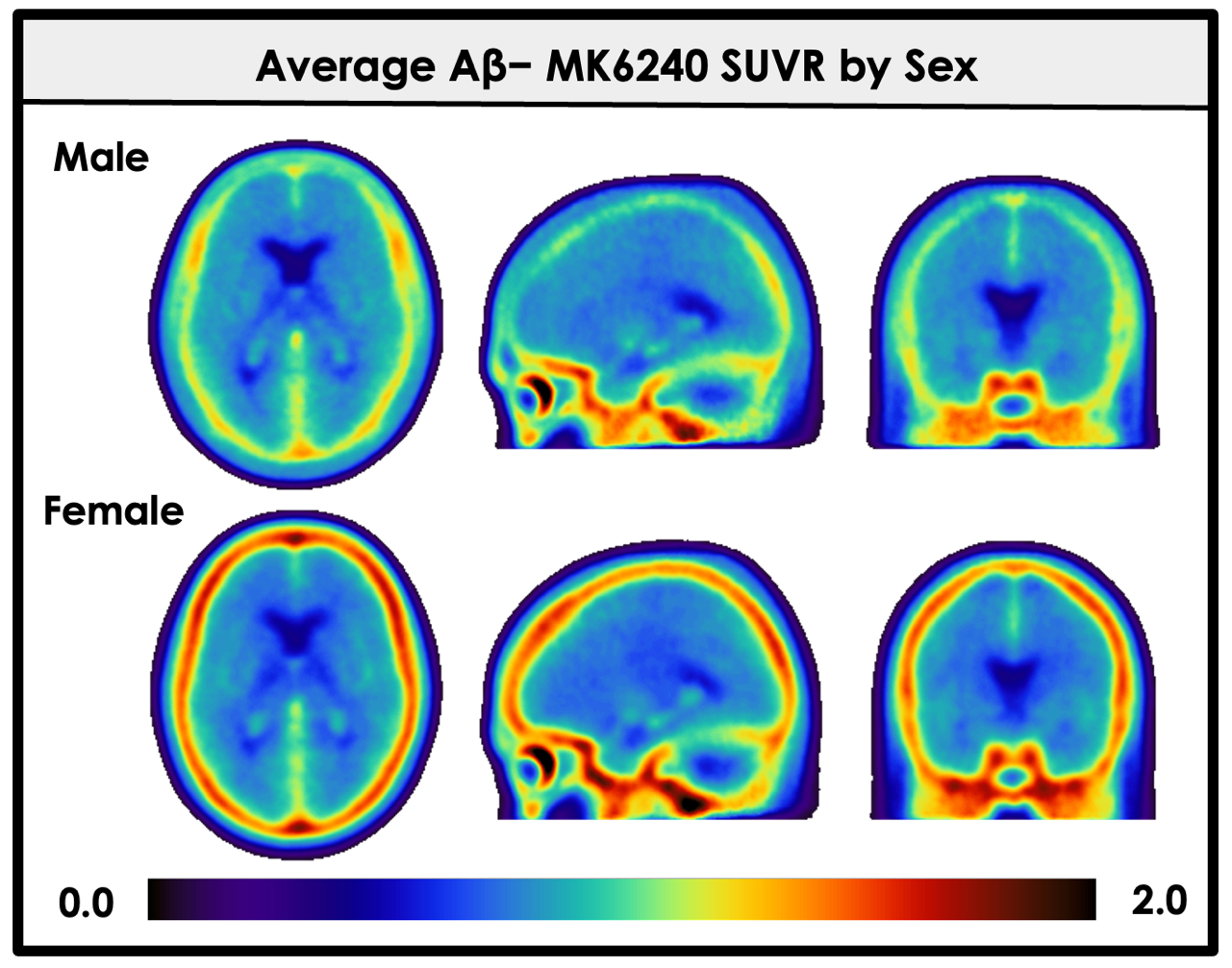


**Figure S4: MK6240 signal in the meninges is higher in women compared to men.** 100 Aβ- men and 100 Aβ- women were randomly selected from the present study cohort. Averaged template space MK6240 SUVR images for each of these groups show visually distinct differences in the intensity of MK6240 signal in the meninges by sex. Colorbar represents mean SUVR.

**Supplementary Figure S5**


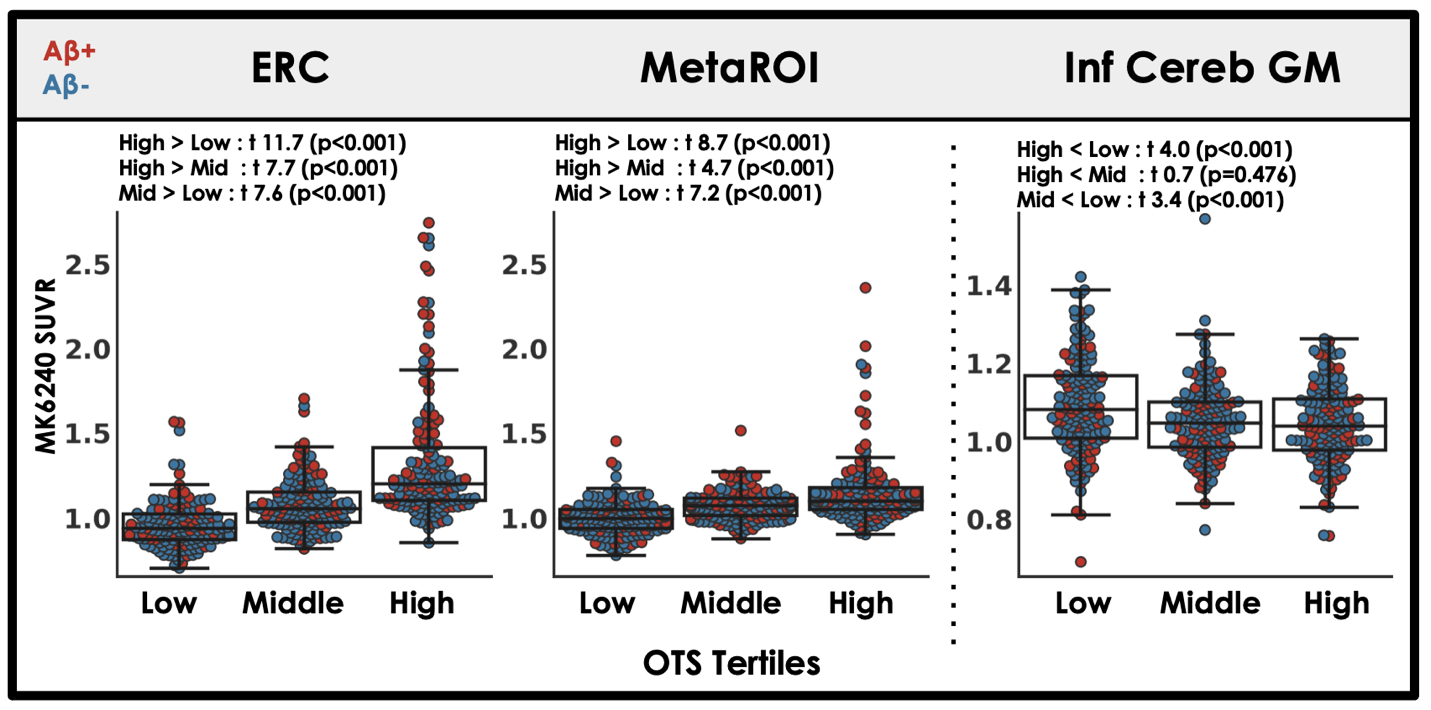


**Figure S5**: **Target Regions SUVRs show step-wise changes related to OTS ROI SUVR tertiles.** Regional [18F]MK6240 OTS ROI SUVR (see Fig. 1B) divides the cohort into three groups for each panel: low, middle, and high OTS. SUVR values within three target regions of interest are plotted by OTS tertile. Student's t-test describes the difference in hemispheric GM intensity between OTS tertile groups. ERC and MetaROI SUVRs were normalized to the inferior cerebellar GM. Inferior cerebellar GM SUVR was normalized to Braak V/VI. Individual points are labeled by Aβ status: positive (red) and negative (blue)

**Supplementary Figure S6**

**
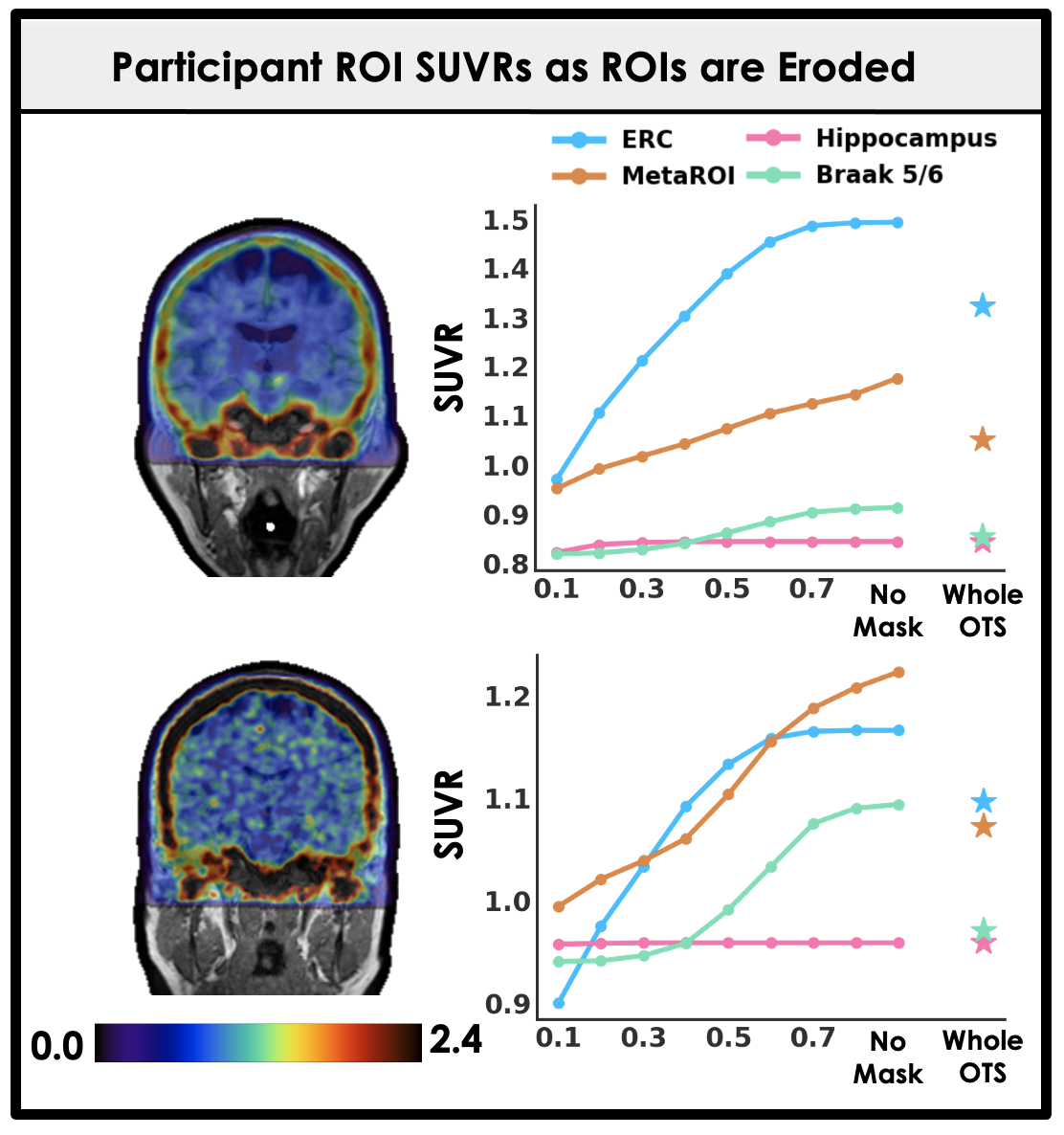
**

**Figure S6: Single participant examples illustrate OTS effects on target region quantification.** Two participant examples are shown. On the left is their MK6240 scan overlaid on their MRI. Colorbar represents SUVR. On the right, the SUVR of 4 target regions are plotted at increasingly aggressive masking thresholds. Importantly, SUVR in regions with adjacent OTS such as ERC and the temporal MetaROI continue to decrease as more and more of the target region is eroded away from the OTS.

**Supplementary Figure S7**

**
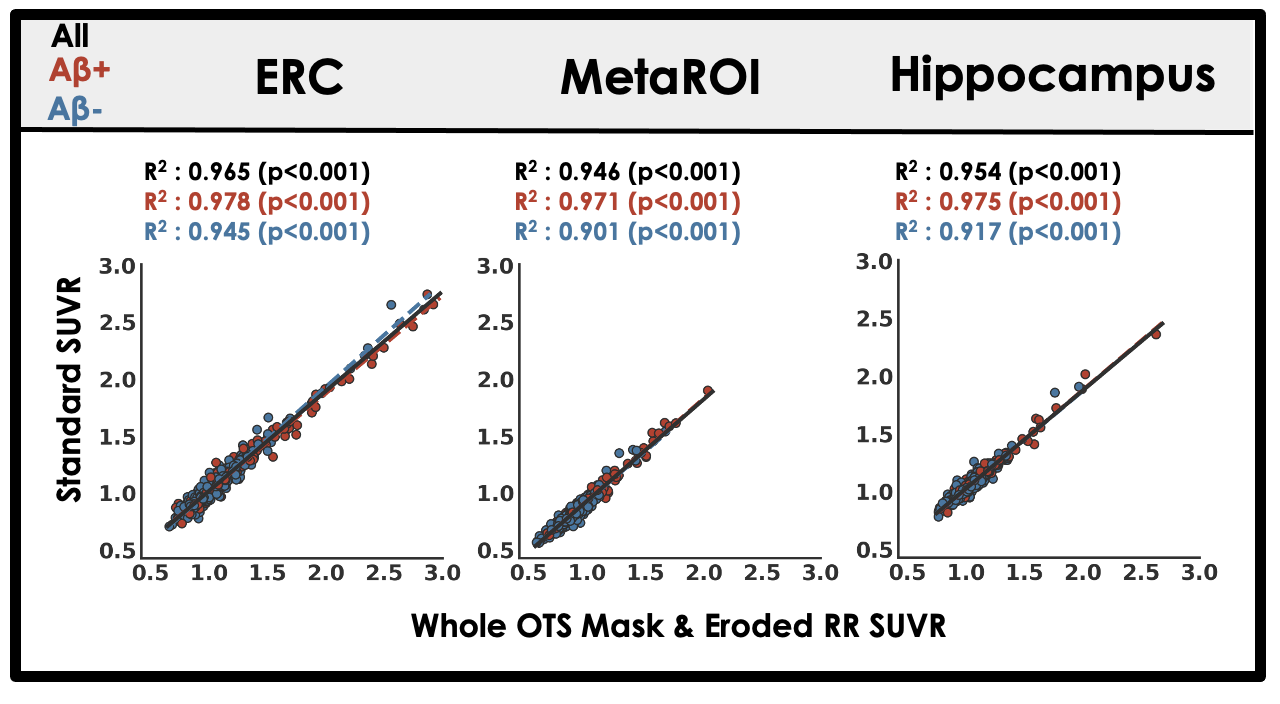
**

**Figure S7: Relationship between standard MRI-guided PET quantification and theoretically “optimized” quantification.** SUVRs generated after removing OTS from target regions and the reference region are highly correlated with standard processing SUVRs.
